# Direct comparison of CMR dobutamine stress wall motion and perfusion analysis with adenosine perfusion in patients after bypass surgery

**DOI:** 10.1186/1532-429X-13-S1-P100

**Published:** 2011-02-02

**Authors:** Christoph Klein, Rolf Gebker, Sebastian Kelle, Kristof Graf, Stephan Dreysse, Bernhard Schnackenburg, Eckart Fleck

**Affiliations:** 1 German Heart Institute Berlin, Berlin, Germany; 2 Philips Clinical Science, Hamburg, Germany

## Introduction

Dobutamine and adenosine stress are the two methods most often used in CMR for the diagnosis of ischemia. Both methods have demonstrated good results in patients after coronary artery bypass graft (CABG). However, a direct comparison has not been examined.

## Purpose

Comparison of the diagnostic accuracy of dobutamine wall motion analysis (DSMR) and perfusion (DSPERF) with adenosine perfusion (APERF)

## Methods

One hundred nine patients (93 men, 65±8 years, BMI 28.7±3.3) after CABG underwent CMR imaging on two appointments. 1) LV-function, DSMR (10-40µg/kg/min + 2mg atropine if needed until target heart rate) imaging (SSFP) of 3 short axis (apical, medial, basal) and 3 long axis views at each stress level. Perfusion imaging of 3 short axis (SSFP, TR/TE 2.8 ms/1.4 ms, FA 50°, SENSE-factor 3.0) were acquired every second heart beat during dobutamine stress at maximal stress level with a contrast bolus of 0.1mmol/kg Gd-DTPA. Late gadolinium enhancement (LGE) imaging (3D inversion recovery technique, TE/TR 2.8/6.6, FA 15°) 10min after additional 0.1mmol/kg Gd-DTPA. 2) Adenosine and rest perfusion (140 µg/min/kg body weight) (SSFP, TE/TR 2.7/1.4, FA 50°, 3 slices per heart beat) using a 0.05mmol/kg contrast bolus of Gd-DTPA. Images were analyzed visually using the standard 16 segment model. A developing wall motion abnormality or stress induced perfusion defect (larger, if LGE is present) in >1 segment was defined as pathological. Invasive coronary angiography served as the reverence. Significant stenosis was defined as >50% in a bypass graft or a native vessel > 2mm diameter in areas without >75% transmural LGE.

## Results

**Figure 1:**
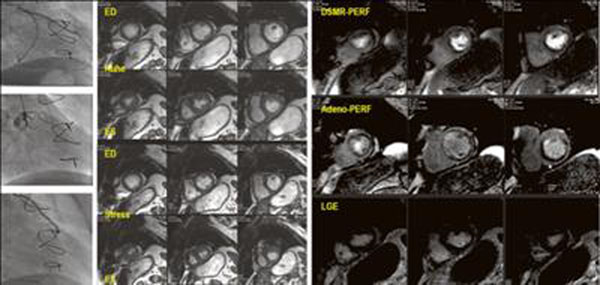


Prevalence of angiographically significant stenosis was in 63% of patients. Surgery was performed 9.5±6.6 years before CMR. Stent placement after CABG was performed in 68 patients. Ejection fraction was 48±8%. LGE was present in 68% of patients. Sensitivity, specificity and diagnostic accuracy are shown in table 1. Figure 1.

**Table 1:** Diagnostic accuracy

	Sensitivity	Seceficity	Diagnostic accuracy
DSMR	88%	96%	93%
DSPERF	86%	73%	79%
DSMR + DSPERF	95%	73%	83%
APERF	75%	91%	85%

## Conclusions

DSMR was the best test for the detection of ischemia in patients after bypass surgery and outperforms adenosine stress perfusion. The addition of DSMR perfusion increases sensitivity, but decreases diagnostic accuracy.

Caption for image: Angiographically ischemia in the inferior and lateral wall. Wall motion abnormalities in the lateral wall. Perfusion defect (dobutamine and adenosine) is larger extending to the inferior wall and apical anterior. No LGE present.

